# Aryl Hydrocarbon Receptor Inhibition Restores Indoxyl Sulfate-Mediated Endothelial Dysfunction in Rat Aortic Rings

**DOI:** 10.3390/toxins14020100

**Published:** 2022-01-26

**Authors:** Cindy Nguyen, Amanda J. Edgley, Darren J. Kelly, Andrew R. Kompa

**Affiliations:** Department of Medicine, St Vincent’s Hospital, University of Melbourne, Fitzroy 3065, Australia; nguyencindy@live.com.au (C.N.); aedgley@unimelb.edu.au (A.J.E.); darrenjk@unimelb.edu.au (D.J.K.)

**Keywords:** aryl hydrocarbon receptor, indoxyl sulfate, endothelial dysfunction, chronic kidney disease, cardiovascular disease, oxidative stress

## Abstract

The uremic toxin indoxyl sulfate (IS), elevated in chronic kidney disease (CKD), is known to contribute towards progressive cardiovascular disease. IS activates the aryl hydrocarbon receptor (AhR) mediating oxidative stress and endothelial dysfunction via activation of the CYP1A1 pathway. The present study examines AhR inhibition with the antagonist, CH223191, on IS-mediated impairment of vascular endothelial function and disruption of redox balance. The acute effects of IS on endothelium-dependent relaxation were assessed in aortic rings from Sprague Dawley rats exposed to the following conditions: (1) control; (2) IS (300 μM); (3) IS + CH223191 (1 μM); (4) IS + CH223191 (10 μM). Thereafter, tissues were assessed for changes in expression of redox markers. IS reduced the maximum level of endothelium-dependent relaxation (Rmax) by 42% (*p* < 0.001) compared to control, this was restored in the presence of increasing concentrations of CH223191 (*p* < 0.05). Rings exposed to IS increased expression of CYP1A1, nitro-tyrosine, NADPH oxidase 4 (NOX4), superoxide, and reduced eNOS expression (*p* < 0.05). CH223191 (10 μM) restored expression of these markers back to control levels (*p* < 0.05). These findings demonstrate the adverse impact of IS-mediated AhR activation on the vascular endothelium, where oxidative stress may play a critical role in inducing endothelial dysfunction in the vasculature of the heart and kidneys. AhR inhibition could provide an exciting novel therapy for CVD in the CKD setting.

## 1. Introduction

CKD affects 10% of the global population, impacting not only health systems but also placing a heavy economic and productivity burden on countries worldwide [1,2]. The risk of cardiovascular disease (CVD) is increased in patients with chronic kidney disease (CKD) [3]. In these patients, the accumulation of non-dialyzable circulating protein-bound uremic toxins (PBUTS) such as the indoxyl sulfate (IS) not only demonstrate an increased CVD mortality [4], but also predict adverse cardiovascular events in patients with chronic heart failure [5]. CKD and elevated IS levels also exacerbate cardiovascular complications such as myocardial hypertrophy, dyslipidemia, chronic inflammation [6], as well as endothelial dysfunction, evidenced by abnormal brachial arterial flow mediated dilatation (FMD) correlated with serum IS levels [7,8]. Other studies have observed that narrowing of the microvasculature in patients with CKD is associated with decreased estimated glomerular filtration rate [9]. Furthermore, animal studies have reported that IS, whether increased in a model CKD or administered exogenously, contributes to impaired endothelium-dependent vasorelaxation [10,11].

IS is derived from tryptophan metabolism that progressively accumulates in the serum of advancing CKD stages 2–5; its high binding-affinity to albumin impedes its clearance even following dialysis [4,12]. Previous studies have identified IS as an endogenous agonist at the aryl hydrocarbon receptor (AhR), a cytosolic ligand-dependent transcription factor that is constitutively expressed and involved in numerous biological processes such as oxidative stress, inflammation, vascular remodeling, and atherogenesis [13,14]. IS-mediated AhR activation induces the enzyme, cytochrome P450 1A1 (CYP1A1) in endothelial cells and this is associated with increased production of reactive oxygen species (ROS) [14,15]. Furthermore, increased expression of CYP1A1 in the blood of patients with CKD has been observed [16]. Inhibition of IS-mediated AhR activation with selective antagonists such as the 2-methyl-2H-pyrazole-3-carboxylic acid (2-methyl-4-o-toly-lazo-phenyl)-amide (CH223191) [17] have been reported to reverse IS-induced inflammation and ROS production to control levels in endothelial cells [14,15].

AhR is known to play a role in maintaining genomic redox balance by activation of phase I xenobiotic and phase II anti-oxidative response elements (ARE) Activation of AhR triggers a cellular detoxification system, which is divided into phase I enzymes such as CYP1A1 involved in ROS production. Continued ROS generation leads to its interaction with the anti-oxidant transcription factor nuclear factor erythroid 2-related factor (Nrf2), additionally, AhR activation also stimulates Nrf2 [18,19]. Nrf2 together with the transcription factor proto-oncogene c-Maf binds to the ARE-inducing expression of phase II anti-oxidative enzymes NAD(P)H:quinone oxidoreductase (NQO1) and glutathione S-transferase (GSTA1/2). CYP1A1 and NRF2 play a homeostatic role in maintaining a balance between pro- and anti-oxidants [18,19].

Treatment options that limit the damaging effects of PBUTs such as IS are limited. The oral adsorbent AST-120, which removes uremic toxin precursors from the gut, such as indole, reduces the formation and circulating levels of IS and other indole-derived PBUTs. AST-120 has been used in small clinical trials with some success [20,21,22], which has seen it approved in several Asian countries. However, the large phase III clinical trials EPPIC-1 and -2 (Evaluating Prevention of Progression in CKD) demonstrated that AST-120 did not meet its primary endpoint [23]. This raises the need for alternative strategies that can prevent the mechanism of action of IS. In vitro, AhR has been shown to be an effective target whose inhibition can block the downstream actions of IS and potentially other tryptophan metabolites [13,14] and may provide therapeutic utility in reducing CVD risk in CKD and alleviate its progression.

While most in vitro studies assessing AhR inhibition use siRNA or small molecule inhibitors such as the pure AhR antagonist CH223191 [17], no ex vivo studies have been performed evaluating the effect of this antagonist on endothelial relaxation to determine ‘proof of principle’. The aim of the present study was to assess the effect of AhR inhibition on isolated aortic tissue to prevent IS-induced impairment of endothelial-dependent vascular function by alleviating IS-mediated oxidative stress and CYP1A1 gene expression associated with AhR activation, and furthermore examine the effects on the anti-oxidant system.

## 2. Results

### 2.1. Effect of Aryl Hydrocarbon Receptor Inhibition on IS-Impaired Endothelium-Dependent Relaxation

**Figure 1:**
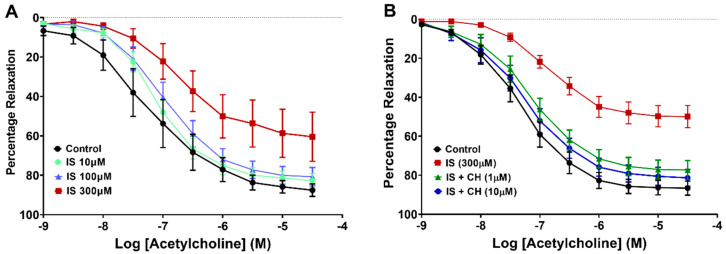
Effect of CH223191 on vascular reactivity. (**A**) Exposure of aortic rings to IS (300 μM) reduced ACh-stimulated endothelium-dependent relaxation (*p* < 0.05; N = 6). (**B**) Preincubation with CH223191 (1 μM and 10 μM) prevented the reduction in IS-induced endothelium-dependent relaxation (*p* < 0.001; N = 10). Data points expressed as mean ± SEM.

Aortic rings from healthy rats were exposed to IS (10–300 μM) to determine the optimal dose of IS causing impairment of endothelial relaxation. IS at 300 μM but not 10 μM or 100 μM significantly reduced endothelium-dependent relaxation (R*max*) to acetylcholine (ACh) compared to control by 32% (*p* < 0.05; Figure 1A), and this concentration was used in further experiments. In subsequent experiments in the presence of the AhR inhibitor, CH223191 (1 and 10 μM), IS-induced impairment of R*max* (42%) was prevented (*p* < 0.001, Figure 1B, Table 1). In IS-only stimulated aortic rings, the potency of ACh (-pEC_50_) was reduced relative to control (*p* < 0.01) and restored in the presence of CH223191 (*p* < 0.05; Table 1). To determine whether the AhR inhibitor alone displayed any intrinsic effect on endothelium-dependent relaxation, aortic rings were incubated with CH223191 alone, and exhibited no effect on R*max* or -pEC_50_ values (Table 1, also Supplemental Figure S1).

### 2.2. Effect of IS on the Endothelium and Endothelial Nitric Oxide Synthase

**Figure 2:**
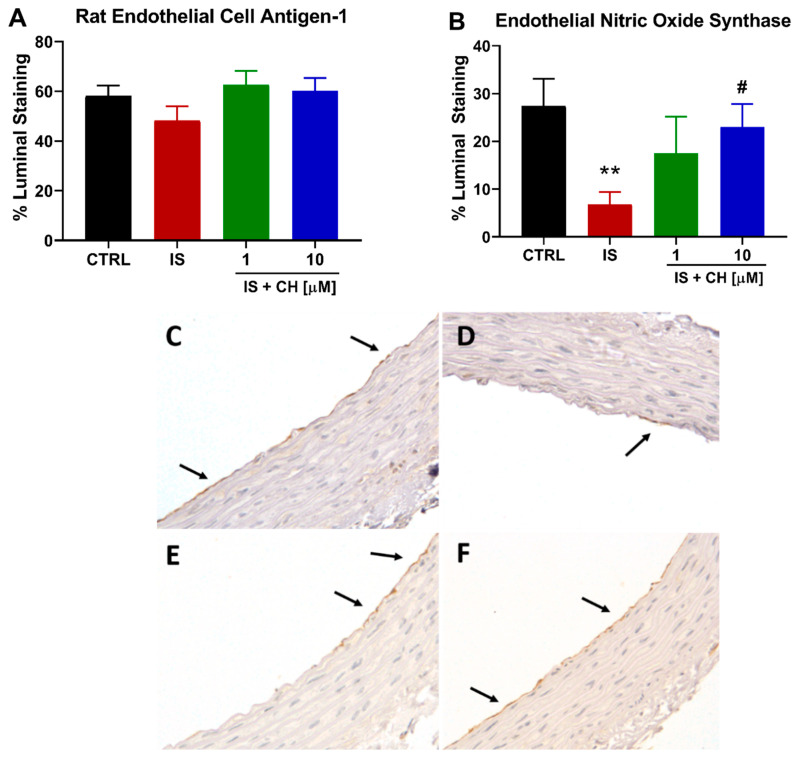
IS reduces eNOS expression without loss of endothelial cells. (**A**) The endothelial cell marker RECA-1 staining in IS-treated aortic rings in the absence and presence of CH223191. (**B**) Endothelial NOS expression reduced in the presence of IS was prevented with CH223191. Representative images of eNOS staining in (**C**) control tissue, (**D**) IS-treated, IS in the presence of (**E**) 1 μM and (**F**) 10 μM CH223191. Arrows indicate positive staining. Data expressed as mean ± SEM from N = 10 experiments. ** *p* < 0.01 compared to control tissue, # *p* < 0.05 compared to IS exposed tissue. Magnification X400.

On completion of the functional relaxation responses to ACh, tissue sections from these aortic rings were analyzed by immunohistochemistry for endothelial cells and endothelial nitric oxide synthase (eNOS) expression. There was no change in endothelial cells as determined by staining for rat endothelial cell antigen-1 (RECA-1) under the four conditions examined (Figure 2A). However, aortic ring sections stained for eNOS demonstrated that IS-exposure significantly reduced eNOS expression by 75% of the endothelial luminal surface (*p* < 0.01), whereas in the presence of CH223191 this reduction was prevented in the presence of 10 μM CH223191 (Figure 2B–F; *p* < 0.05).

### 2.3. Effect of IS and CH223191 on Pro- and Anti-Oxidative Gene Expression

**Figure 3:**
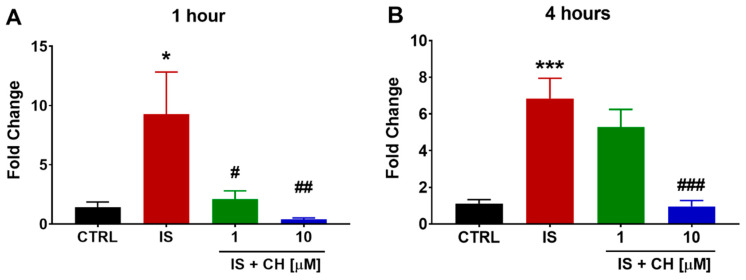
Effect of IS and AhR inhibition on CYP1A1 gene expression. Exposure of aortic rings to IS increased CYP1A1 gene expression at (**A**) 1 and (**B**) 4 h. In the presence of CH223191 IS-induced CYP1A1 expression was prevented (**A**,**B**). Data expressed as mean ± SEM from N = 6 experiments. * *p* < 0.05, *** *p* < 0.001 compared to control tissue, # *p* < 0.05, ## *p* < 0.01, ### *p* < 0.001 compared to IS exposed tissue.

IS-induced activation of AhR is known to increase CYP1A1. We found IS (300 μM) significantly increased CYP1A1 expression in aortic rings after 1 and 4 h by nearly nine- and seven-fold, respectively, compared to control conditions (*p* < 0.05, Figure 3). CH223191 at 1 and 10 μM reduced IS-mediated CYP1A1 gene expression after 1 h IS stimulation to similar levels observed in control-exposed rings, whereas after 4 h IS stimulation, only 10 μM CH223191 was able to significantly reduce CYP1A1 levels (*p* < 0.001, Figure 3). On examining the anti-oxidative pathway, IS in the absence and presence of CH223191 failed to induce a change in gene expression of Nrf2, NQO1 and GSTA1/2 (See Supplemental Figure S2).

### 2.4. Effect of IS and CH223191 on Markers of Oxidative Stress and Inflammation

**Figure 4:**
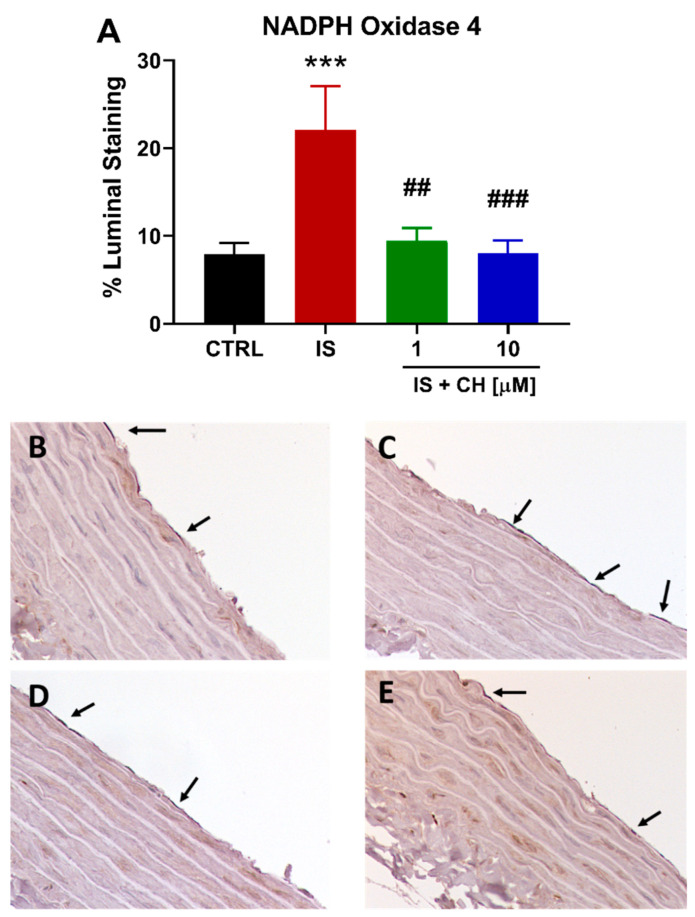
CH223191 prevented IS-induced NOX4 expression. (**A**) Quantitation of NOX4 staining in IS-treated aortic rings in the absence and presence of CH223191. Representative images of NOX4 expression in (**B**) control tissue, (**C**) IS-treated, IS in the presence of (**D**) 1 μM and (**E**) 10 μM CH223191. Data expressed as mean ± SEM from N = 10 experiments. *** *p* < 0.001 compared to control tissue, ## *p* < 0.01, ### *p* < 0.001 compared to IS exposed tissue.

**Figure 5:**
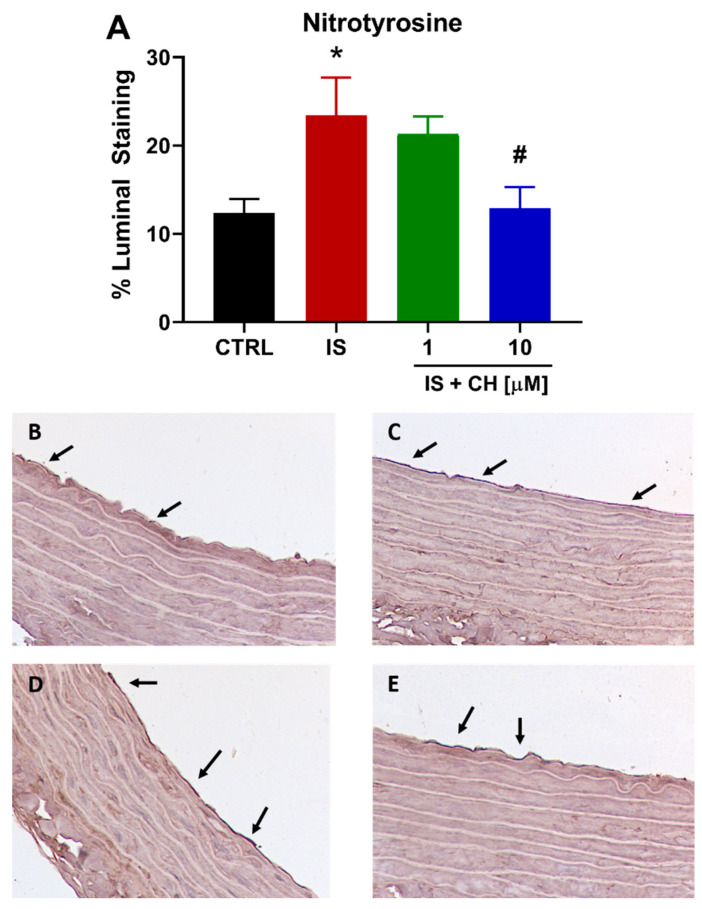
CH223191 prevented IS-induced nitro-tyrosine expression. (**A**) Quantitation of nitro-tyrosine staining in IS-treated aortic rings in the absence and presence of CH223191. Representative images of nitro-tyrosine expression in (**B**) control tissue, (**C**) IS-treated, IS in the presence of (**D**) 1 μM and (**E**) 10 μM CH223191. Data expressed as mean ± SEM from N = 10 experiments. * *p* < 0.05 compared to control tissue, # *p* < 0.05compared to IS exposed tissue.

**Figure 6:**
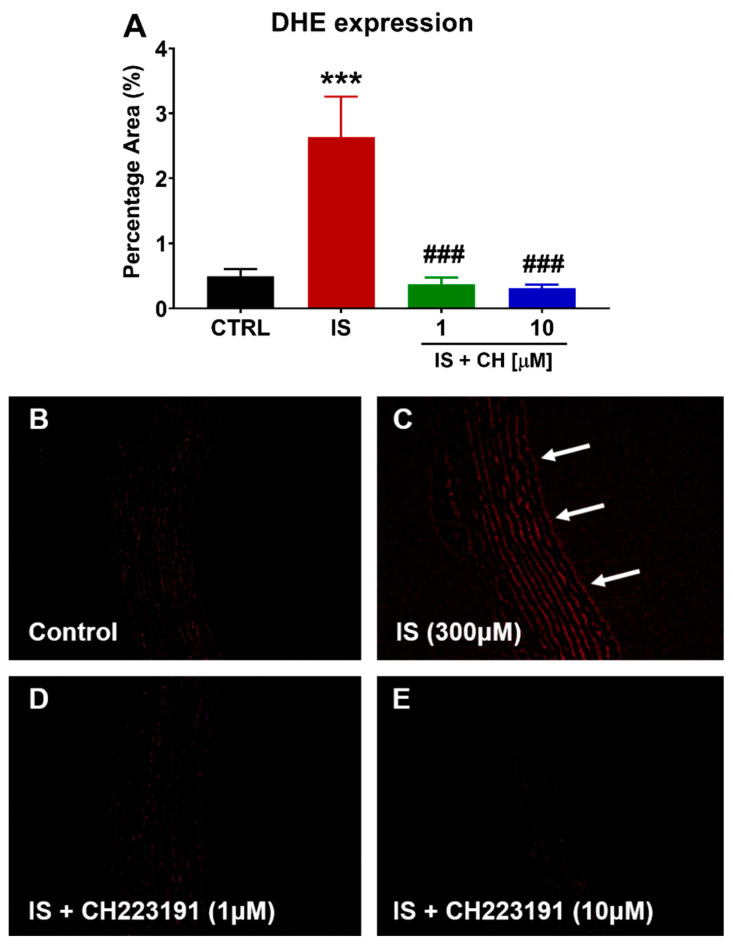
CH223191 prevented IS-induced superoxide expression. (**A**) Quantitation of DHE staining in IS-treated aortic rings in the absence and presence of CH223191. Representative images of superoxide expression in (**B**) control tissue, (**C**) IS-treated, IS in the presence of (**D**) 1 μM and (**E**) 10 μM CH223191. Data expressed as mean ± SEM from N = 10 experiments. *** *p* < 0.001 compared to control tissue, ### *p* < 0.001 compared to IS exposed tissue. Magnification ×200.

To further examine changes in redox balance, tissues from the functional experiments were analyzed to examine changes in oxidative stress markers, namely nicotinamide adenine dinucleotide phosphate (NADPH) oxidase 4 (NOX4), superoxide expression and nitro-tyrosine. Tissues exposed to IS demonstrated a 180% increase in NOX4 expression (Figure 4; *p* < 0.001). In the presence of CH223191 (1 and 10 μM), NOX 4 expression was significantly reduced compared to IS (Figure 4; *p* < 0.001). IS exposure increased expression levels of nitro-tyrosine, a marker of cell damage and inflammation staining by 90% compared to control (Figure 5; *p* < 0.05), whereas in the presence CH223191 (10 μM), nitro-tyrosine expression was reduced back to control levels (Figure 5; *p* < 0.05). Expression of the reactive oxygen species molecule, superoxide, was determined by dihydroethidium (DHE) staining. IS exposure increased DHE staining by 430% compared to control (Figure 6; *p* < 0.001), whereas in the presence CH223191 at both doses, superoxide expression was reduced back to control levels, as shown in Figure 6; (*p* < 0.001).

**Figure 7:**
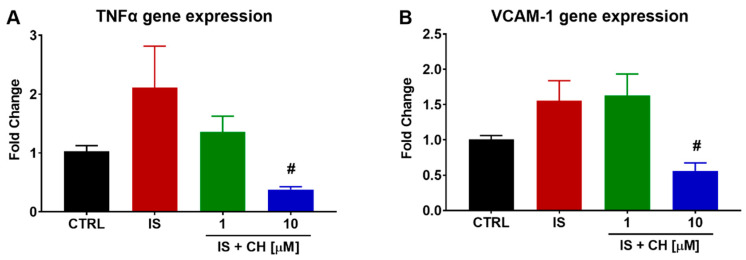
Effect of IS and AhR inhibition on inflammatory gene expression. Exposure of aortic rings to IS non-significantly elevated (**A**) TNFα and (**B**) VCAM-1 gene expression at 4 h. In the presence of CH223191, TNFα and VCAM-1 expression was prevented. Data expressed as mean ± SEM from N = 6 experiments. # *p* < 0.05 compared to IS exposed tissue.

Gene expression of inflammatory markers was also examined in aorta stimulated with IS in the absence and presence of either CH223191. IS produced a non-significant increase of 111 and 55% in tumour necrosis factor α (TNFα) and vascular cell adhesion molecule-1 (VCAM-1) gene expression, respectively, at 4 h. In the presence of CH223191, expression levels of both genes were significantly reduced, as shown in Figure 7 (*p* < 0.05).

## 3. Discussion

Increased CYP1A1 and ROS production induced by AhR activation plays an integral role in the impairment of endothelium-dependent vasodilatation [24,25]. The results of the present study suggest that blockade of the AhR with CH223191 alleviated IS-mediated pro-oxidative CYP1A1 gene expression and ROS production in the aorta and preserved eNOS expression and the endothelium-dependent vasodilator response to ACh. Impairment of endothelium-dependent vasodilatation following IS exposure has previously been observed in the clinical setting in CKD patients, furthermore, lowering IS levels in the circulation with AST-120 improved FMD in CKD patients [8] and improved endothelial response in the microvasculature of end-stage renal disease patients using iontophoresis [26]. Similarly, in-animal models of CKD, treatment with AST-120 restored endothelium-dependent relaxation in aortic rings [11]. Together, these findings indicate that IS is involved in the impairment of endothelial-dependent function in microvascular and conduit vessels. A potential limitation to this study is that experiments were performed in the absence of plasma proteins such as albumin, hence the concentration of free IS used for these acute studies may be higher than that observed in CKD patients, however the purpose of the current study was to investigate mechanisms of IS-induced vascular function

The vascular endothelium regulates vessel tone in conduit and resistance arteries via several factors with nitric oxide (NO) thought to be the main mechanism affected by uremia [8,10,11,26,27,28]. Previous studies have demonstrated reduced endothelial nitric oxide synthase (eNOS) and NO production in IS-stimulated endothelial cells is associated with increased ROS through NADPH oxidase activation, as well as inhibition of anti-oxidant systems [8,10,14,15]. Furthermore, CKD rats treated with AST-120 reported a restoration in NO and eNOS expression, and lower serum IS levels [29]. In addition, genetic deletion of AhR reduces pulse wave velocity compared to wild type mice, indicating enhanced vessel elasticity, which is accompanied by an increase in NO content and eNOS activity [30]. Results from these previous studies are in agreement with those observed here, where an increase in endothelial NOX4, nitrotyrosine and superoxide expression and a concomitant reduction in eNOS was observed following IS stimulation. Moreover these changes in the endothelium were prevented in the presence of CH223191. Previous studies have examined endothelium-independent relaxation in the aorta in the presence of IS using the NO donor sodium nitroprusside; however, no change in response was observed after even after four days incubation with 1mM IS when compared to control conditions [11], hence this relaxation was not investigated in the current study.

Activation of AhR and its downstream pathway have been reported to play a role in endothelium-dependent vascular dysfunction, hypertension, and cardiac hypertrophy inducing CYP1A1 expression and increasing ROS in the vasculature and heart [24,25]. Studies in CYP1A1 knock-out mice reported that AhR activation prevented the increase in superoxide production in these tissues and restored endothelial relaxation [25]. Moreover, AhR knockout mice, or pharmacological inhibition of the receptor with α-naphthoflavone (α-NF), also a partial agonist at the AhR, in diabetic nephropathy resulted in reduced oxidative stress [31]. In line with these observations, studies of AhR activation of human umbilical vein endothelial cells (HUVECs) have similarly demonstrated an elevation in IS-induced oxidative stress with concomitant reduction in NO, and increased CYP1A1 expression, which were inhibited by AhR inhibition, or prevented from entering the cell by inhibition of the organic anion transporter probenecid [8,15].

Increased oxidative stress can be caused by an imbalance between pro- and anti-oxidative states. On examining AhR induced Nrf2-induced activation of the ARE, we found no change in the expression of anti-oxidant enzymes. Previously, Bolati et al. have shown that increased IS down-regulates Nrf2 gene expression in HK2 cells, furthermore in a rat model of CKD with elevated serum IS, reduced Nrf2 and NQO1 protein expression were attenuated following treatment with AST-120 [32]. Our results suggest that the phase II of the genomic pathway may not yet be activated considering the acute 4 h exposure of IS used in this study. Future evaluation of these anti-oxidant mechanisms are warranted utilizing a longer time course of IS-induced AhR activation.

Indole-3 acetic acid (IAA) is another AhR agonist and PBUT, which is progressively increased in the circulation of CKD patients and predictive of mortality and major adverse cardiovascular events, serum levels of this molecule were correlated with markers of inflammation and oxidative [33]. In addition, IAA stimulation of HUVECs resulted in increased CYP1A1 and cyclo-oxgenase-2 (COX-2) gene expression and increased COX-2 protein and ROS production. In the presence of CH223191, these changes in inflammation and oxidative stress were significantly reduced or abolished [33].

IS-induced inflammatory responses are known to result in the expression of adhesion molecules inflammatory cytokines, and chemokines [11,15,34] that contributes to the pathogenesis of vascular dysfunction. CH223191 has been reported to inhibit IS-induced monocyte chemo-attractant protein-1 (MCP-1) expression in HUVECs [15], thus attenuating vascular inflammation. In addition, siAhR-transfected HUVECs suppressed IS-enhanced vascular inflammation (mediated by TNFα), leukocyte adhesion, and E-selectin gene expression [34]. Furthermore, IS exposure (1 mM) of aortic rings isolated from healthy animals for up to four days, not only reduced endothelium-dependent relaxation, but also reduced CD31 staining and increased vascular cell adhesion molecule-1 (VCAM-1) and intracellular adhesion molecule-1 (ICAM-1) expression in the aorta [11]. By the same token, aortae harvested from CKD mice at ten weeks showed reduced endothelium-dependent relaxation, this was further reduced when incubated with IS. These changes were accompanied by a reduction in CD31 staining and increased ICAM-1 and VCAM-1 staining. Moreover treatment of CKD animals with AST-120 improved endothelium-dependent relaxation and reduced adhesion marker expression toward control levels [11]. Our results examining the short term effect of IS with and without AhR inhibition on VCAM-1 expression is consistent with previous findings outlined above. Interestingly at 4 h following IS stimulation TNFα gene expression appeared to be on the rise, a finding that we have previously demonstrated at 18 h in human aortic endothelial cells, THP-1 cells, rat cardiac myocytes and fibroblasts, and rat renal mesangial cells [35,36,37]. Others have similarly reported increased TNFα expression in monocyte and macrophage cell lines [38,39]. In macrophages, this is thought to involve cross talk between AhR, nuclear factor κB (a central mediator in inflammation), and suppressor of cytokine signaling 2 [39]. However, the direct inhibitory effect of CH223191 on IS-induced TNFα gene expression in the vasculature, to our knowledge, has not previously been reported and this may be a new mechanism for future investigation into IS-induced vascular inflammation.

AhR is ubiquitously expressed [40], suggesting that its inhibition may play a role in attenuating adverse effects in many tissues associated with CKD. We have previously shown that IS induces cardiac and renal collagen synthesis in cardiac fibroblasts and renal mesangial cells as well as myocyte hypertrophy in cardiac myocytes and inflammatory responses in THP-1 cells [35]. Furthermore, in models of CKD and myocardial infarction where serum IS and cardiac and renal fibrosis is increased, AST-120 treatment was shown to reduce these effects [41,42,43]. Ichii et al. (2014) demonstrated chronic IS administration for eight weeks resulted in progressive kidney and vascular disease, observed by increased fibrosis, tubular atrophy, urinary albumin/creatinine ratio, as well as podocyte injury with concomitant increased in CYP1A1 expression, indicative of AhR activation [44]. In the diabetic kidney, AhR is thought to play a role in fibrosis, inflammation, and oxidative stress, increasing expression of trichrome staining, α-smooth muscle actin, fibronectin, serum prostaglandin E_2_ (PGE_2_), serum advanced glycation end products, COX-2, F4/80 macrophage infiltration, NOX activity, and the marker of oxidative damage 8-hydroxydeoxyguanosine. Increased expression of these markers was prevented in diabetic AhR knockout mice and diabetic wild type mice treated with the AhR antagonist α-NF [31]. In addition, in vitro studies by Lee et al. (2016) showed that whilst stimulation of rat and mouse mesangial cells and human kidney tubular cells with N-ε-carboxymethyl-lysine (CML), a major advanced glycation end product, increased expression of AhR, collagen IV, connective tissue growth factor, fibronectin, and PGE_2_, this expression was prevented in cells transfected with shRNA-AhR, or pharmacologically treated with α-NF [31].

It is clear AhR plays an important role in the pathology of cardiovascular and renal disease in the setting of CKD, offering a new possibility for therapeutic intervention. With results from the EPPIC trials reporting a neutral outcome with AST-120 [23], other strategies are required to mitigate the damaging effects of PBUTs in CKD. Given the abundance of data indicating the benefit of AhR inhibition, in vivo, in vitro, or ex vivo, whether genetic or pharmacological, an in vivo proof of principle study examining CH223191 in a clinically relevant model of CKD that shows efficacy would be required to progress this class of drug to the next stage.

In conclusion, the present study demonstrates that IS-mediated impairment of endothelial-dependent vasodilatation is associated with induced oxidative stress, involving an increase in CYP1A1 expression. Reversal of these effects by inhibition of the AhR with CH223191 may provide therapeutic utility in maintaining endothelial function in the CKD population. Potentially, AhR antagonists may be used as an adjunct therapy in addition to standard-of-care treatment in CKD patients and attenuate the progression of CVD risk in this population.

## 4. Materials and Methods

### 4.1. Vascular Reactivity

Regarding the effect of IS in the absence and presence of the AhR antagonist, CH223191 was assessed for endothelial function. Briefly, 10–14 week old male Sprague-Dawley rats were anaesthetized with Lethbarb (Troy Laboratories, Australia) and the descending thoracic aorta harvested as described previously [36]. Aortae were cleaned and dissected into four 5 mm rings and carefully placed between 2 horizontal stainless steel supports with one end connected to a micrometer for adjusting tension. Tissues were submerged into oxygenated tissue baths containing Krebs-Henseleit buffer (119 mM NaCl, 4.7 mM KCl, 1.17 mM MgSO_4_.7H_2_O, 25 mM NaHCO_3_, 1.18 mM KH_2_PO_4_, 11 mM D-glucose, 0.03 mM EDTA, 2.0 mM CaCl_2_) [11], and randomly allocated to the following conditions: (1) Control; (2) IS (300 μM); (3) IS + CH223191 (1 μM); (4) IS + CH223191 (10 μM). Tissues were stabilized under 2 g of baseline tension and washed 5 times with Krebs buffer every 5 min. Viability and contractile responses were assessed with (80 mmol/L) and allowed to reach maximum contraction prior to a 3 × 5 min washout period with buffer.

Tissues placed in Krebs-Henseleit buffer were preincubated with vehicle (0.1% DMSO for conditions 1 and 2) or the AhR antagonist CH223191 (1 or 10 μM for conditions 3 and 4, respectively) for 2 h followed by a 1 h incubation with IS (300 μM, conditions 2–4, the control condition received 120 μL of distilled water). Aortic rings were then preconstricted with phenylephrine (PE, 30 μM), where upon attaining maximum constriction endothelium-dependent vascular function was assessed by a dose-response relaxation curve to acetylcholine (ACh; 1 nM-30 μM). The change in tension was obtained via isometric transducers (FT03C, Grass Instruments, Quincy, MA, USA), amplified (Unicor Instruments, Box Hill, Vic, Australia) and recorded using data acquisition hardware (PowerLab 4sp, ADInstruments, Castle Hill, NSW, Australia) and Chart software (ADInstruments v5.6). The resulting relaxation with each dose of ACh was expressed as a percentage reduction of the maximum contraction obtained with phenylephrine for each condition. Animal experiments were approved by the Animal Ethics Committee of St. Vincent’s Hospital, AEC 006/17.

### 4.2. Gene Expression Studies

Thoracic aortic rings from separate animals were isolated as described above and incubated with Dulbecco’s Modified Eagle’s Medium (DMEM) supplemented with antibiotics (1%) in 5% CO_2_ at 37 °C. Under conditions 1–4 described above, aortic rings were incubated with CH223191 for 2 h followed by the addition of IS for 4 h. RNA was extracted using the Trizol extraction method according to manufacturer’s instructions, followed by DNase treatment and reverse transcription. PCR was performed using SYBR green and sequence-specific primers for rat CYP1A1, Nrf2, NQO1, GSTA1/2, TNFα, and VCAM-1 (see Supplemental Table S1 for primer sequences), using a QuantStudio 7-Flex Real-Time PCR system (Applied Biosystems). Analysis of the relative change in CYP1A1 mRNA expression was conducted using the comparative ∆∆Ct method using the house-keeping gene 18S.

### 4.3. Immunohistochemistry

At the conclusion of the vascular reactivity experiments, aortic rings were immediately fixed in 10% neutral buffered formalin overnight, processed in an automatic tissue processor and paraffin-embedded the following day for immunohistochemistry. LV tissue sections (4 μm) from each experiment were stained for RECA-1 (BioRad #MCA970GA, 1/200 dilution), eNOS (BD Biosciences #610296, 1/200 dilution), NOX4 (Abcam #133303, 1/150 dilution), and nitrotyrosine (Merck #06-284, 1/100 dilution), and detected with diaminobenzimide as previously described [45]. Stained slides were digitally scanned using the Aperio Scanscope scanner with Scanscope Console software (v8, Aperio Technologies) and quantitated for positive luminal staining as a percentage of total lumen perimeter using Imagescope software (v 11.1.2.760, Aperio Technologies).

### 4.4. Assessment of Reactive Oxygen Species

Reactive oxygen species (ROS) were detected in aortic tissue obtained from the functional studies. Tissue sections were stained with dihydroethidium (DHE, 2 μM) for 30 min at 37 °C [46]. A confocal microscope (Nikon A1R, Nikon Instruments Inc., Melville NY, USA) was used to visualize red-fluorescent staining with an emission spectrum of 610 nm at ×20 magnification. The percentage area of DHE staining for was determined using ImageJ (NIH, Bethesda, MD, USA).

### 4.5. Statistical Methods

Effects on endothelial function were compared by a two-way repeated measures’ analysis of variance (ANOVA) with Tukey’s post-hoc comparison to determine the effect of percentage relaxation to each ACh dose. Maximum relaxation (Rmax) and pEC_50_ values for ACh were obtained by non-linear regression followed by a one-way repeated measures ANOVA with Tukey post-hoc comparison. This was used to determine the statistical significance of maximum relaxation (Rmax) and pEC_50_ values for ACh. Similarly, gene expression, and immunohistochemical and DHE staining was compared by a one-way ANOVA with Tukey’s post-hoc comparison.

## Figures and Tables

**Table 1:** CH223191 prevents IS-mediated reductions in ACh response in aortic rings; CH223191 alone has no effect ^1^.

Aortic Ring Exposure Condition (N = 10)	-pEC_50_ (ACh)	R*max* (ACh)
Control	7.34 ± 0.09	86.87 ± 3.52
IS (300 μM)	6.86 ± 0.12 **	50.31 ± 5.63 ***
IS + CH223191 (1 μM)	7.16 ± 0.12 ^#^	77.49 ± 4.82 ^###^
IS + CH223191 (10 μM)	7.26 ± 0.12 ^#^	81.48 ± 4.22 ^###^
Effect of CH223191 alone (N = 3)		
Control	7.31 ± 0.03	95.21 ± 1.16
IS + CH223191 (1 μM)	7.44 ± 0.23	87.34 ± 8.41
IS + CH223191 (10 μM)	7.05 ± 0.23	97.04 ± 1.16

^1^ Data are reported as mean ± SEM. ** *p* < 0.01, *** *p* < 0.001 compared to control conditions; ^#^ *p* < 0.05, ^###^ *p* < 0.001 compared to IS stimulated conditions.

## Data Availability

Data supporting results can be found with the corresponding author.

## Supplementary Materials

The following supporting information can be downloaded at: https://www.mdpi.com/article/10.3390/toxins14020100/s1, Figure S1: Effect of CH223191 alone on vascular reactivity in isolated rat aortic rings; Figure S2: Effect of IS and AhR inhibition on Nrf2 and antioxidant gene expression Table S1: Nucleotide sequences of primers used for gene quantification by real time PCR.
