# Supplementary material for: Aryl Hydrocarbon Receptor Inhibition Restores Indoxyl Sulfate-Mediated Endothelial Dysfunction in Rat Aortic Rings
Source: Toxins (Basel). 2022 Jan 26;14(2):100. doi: 10.3390/toxins14020100 (PMC8878015; doi:10.3390/toxins14020100)
Supplement: Supplementary file 1 [file toxins-14-00100-s001.zip › toxins-1535726-supplementary.pdf]

Supplementary Materials: Aryl Hydrocarbon Receptor Inhibition Restores Indoxyl Sulfate-Mediated Endothelial Dysfunction in Rat Aortic Rings

Cindy Nguyen, Amanda J. Edgley, Darren J. Kelly and Andrew R. Kompa

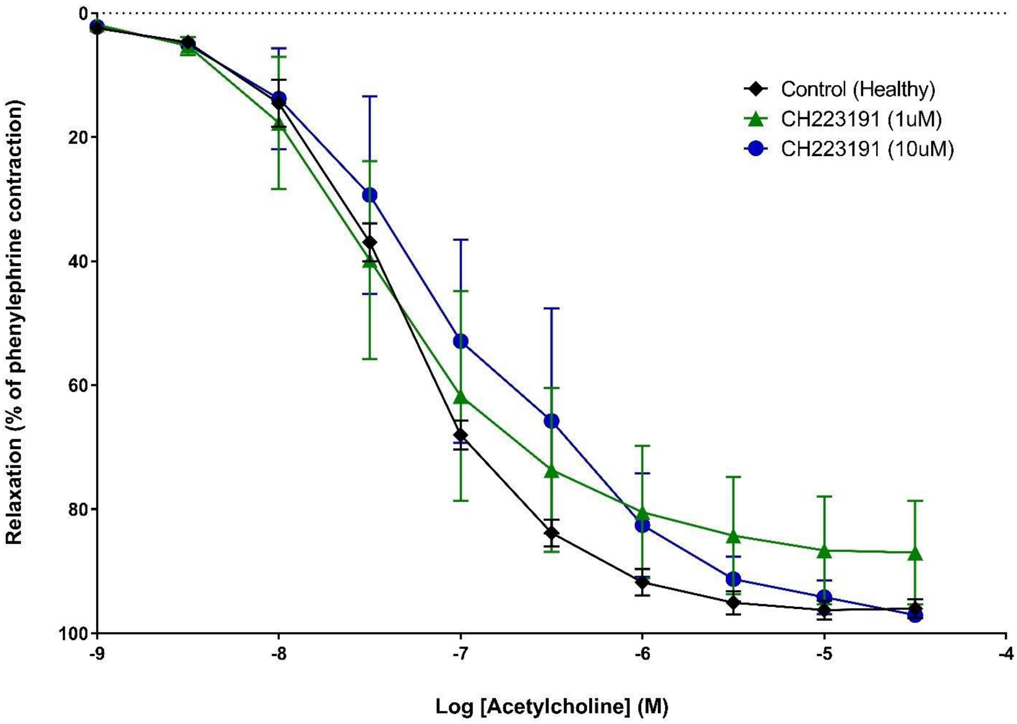

Figure S1. Effect of CH223191 alone on vascular reactivity in isolated rat aortic rings. Dose-response curves to acetylcholine were unaffected in the presence of CH223191 (1 and 10μM) alone compared to control.

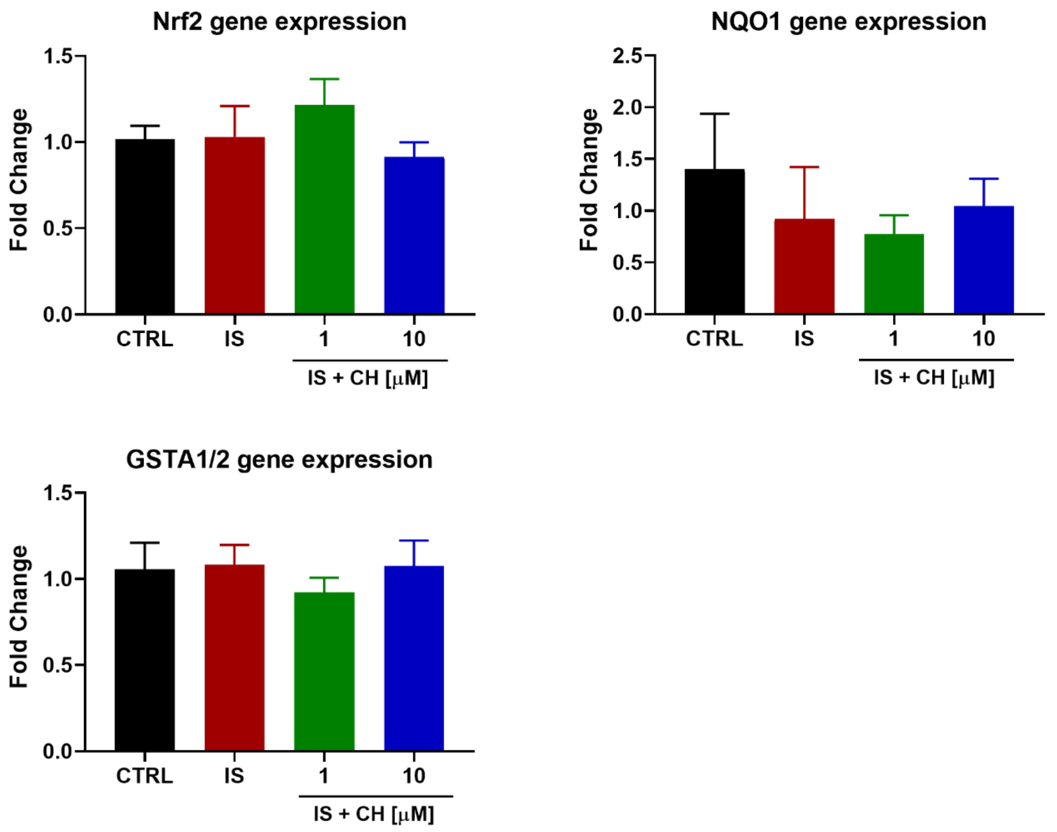

Figure S2. Effect of IS and AhR inhibition on Nrf2 and antioxidant gene expression. Exposure of aortic rings to IS in the absence and presence of CH223191 did not affect gene expression Nrf2, NQO1 and GSTA1/2. Data expressed as mean ± SEM from N=6 experiments.

Table S1. Nucleotide sequences of primers used for gene quantification by real time PCR.

Gene of Interest	Primer Sequence (5′-3′)	
**Rat 18S (House-keeping gene)**	Forward:	TCGAGGCCCTGTAATTGGAA
	Reverse:	CCCTCCAATGGATCCTCGTT
**Rat CYP1A1**	Forward:	CCACAGCACCATAAGAGATACAAG
	Reverse:	CCGGAACTAGTTTGGATCAC
**Rat GSTA1/2**	Forward:	GGAGATTGATGGGATGAAGC
	Reverse:	AACACCTTTTCAAAGGCAGG
**Rat Nrf2**	Forward:	CTCCCAGGTTGCCCACATTC
	Reverse:	CCTGCCAAACTTGCTCCATG
**Rat NQO1**	Forward:	GACATGAACGTCATTCTCTG
	Reverse:	CTAGCTTTGATCTGGTTGTC
**Rat TNFα**	Forward:	GTGATCGGTCCCAACAAGGA
	Reverse:	CGCTTGGTGGTTTGCTACG
**Rat VCAM-1**	Forward:	ACAAAACGCTCGCTCAGATT
	Reverse:	GTCCATGGTCAGAACGGACT
